# Supplementary material for: Droplet Microfluidic Optimisation Using Micropipette Characterisation of Bio-Instructive Polymeric Surfactants
Source: Molecules. 2021 May 31;26(11):3302. doi: 10.3390/molecules26113302 (PMC8197901; doi:10.3390/molecules26113302)
Supplement: Supplementary file 1 [file molecules-26-03302-s001.zip › molecules-1224548-supplementary.pdf]

Supplementary Information

By using polymer surfmers, it is possible to create functionalised particle surfaces that can be tailored for a range of different biological performances. However, the stability of emulsions within the droplet microfluidics apparatus is critical to enable continuous particle droplet formation, and the stability of emulsions without additional surfactants can be observed in Figure S1.

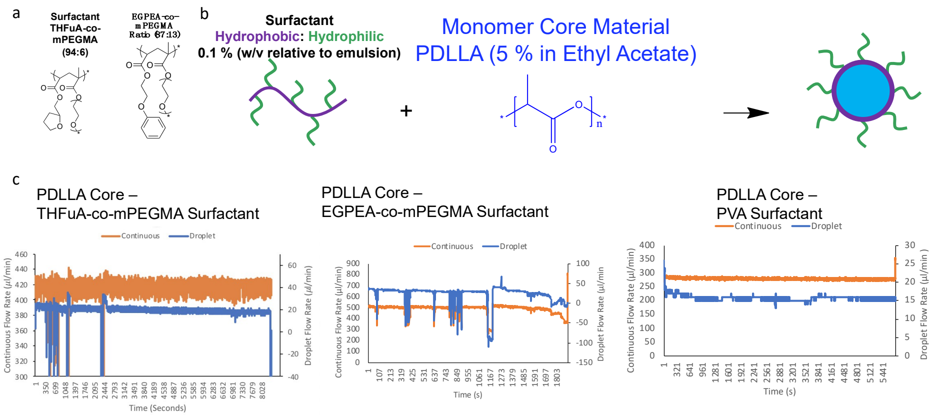

Figure 1. Schematic showing flow diagrams of particles (a) Polymer surfmer structures (b) schematic of emulsion formation using either THFuA-co-mPEGMA, EGPEA-co-mPEGMA or PVA as a surfactant with a 5% w/v PDLLA solution in ethyl acetate and (c)Flow diagrams showing flow instabilities when using either THFuA-co-mPEGMA or EGPEA-co-mPEGMA alone compared to stable flow system observed when using PVA alone.

The stability of emulsions with both THFuA-co-mPEGMA and EGPEA-co-mPEGMA can be shown to be poor with the flow rates of the continuous and dispersed fluids fluctuating. However with the PVA surfactant, the flow rate can be shown to be held constant suggesting better stability of emulsions compared to the surfmer-only systems.

Solvent removal studies were carried out on drops of 1%, 5% and 10% (w/v) PDLLA in ethyl acetate for a range of initial sizes. The size change as ethyl acetate diffused from the drop into the surrounding water can be seen in Figure S2. Micrographs (Figure S2A) depict an example drop, initially 5% PDLLA (w/v) and 39µm radius. The radius for each frame, at 15fps, was measured and plotted in as in Figure S2B. This profile is representative of all drop profiles measured, each following the expected dissolution behaviour as described by the Epstein-Plesset model.

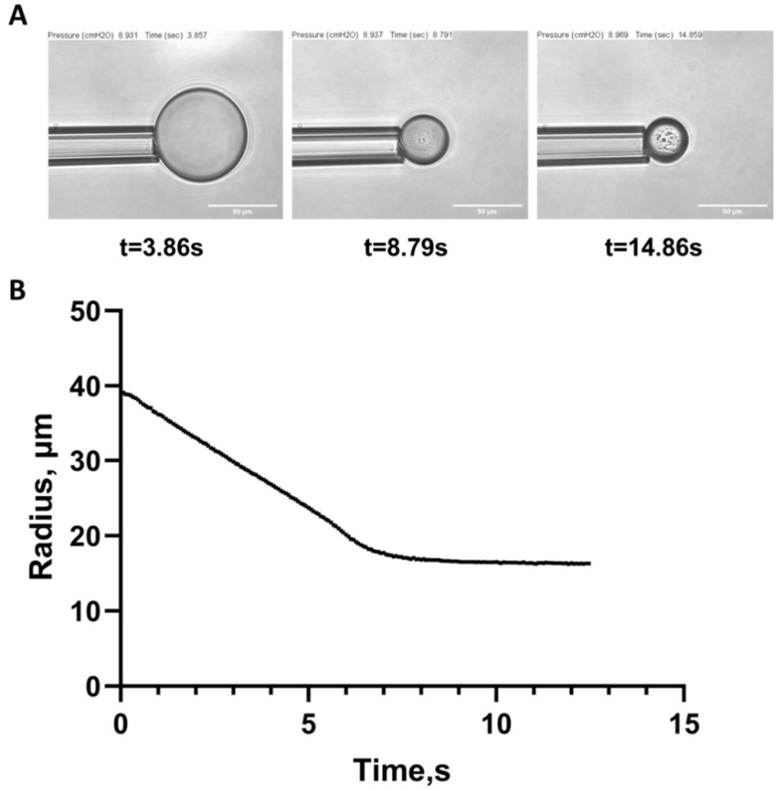

Figure 2. Dissolution of ethyl acetate from a representative drop with initial concentration of 5% PDLLA (w/v) in ethyl acetate as part of the solvent removal studies. (a) micrographs show the drop at 3.86s, 8.79s and 14.86s. (b) the radius is plotted as a function of time, one measurement per frame with a 15fps framerate. All droplet profiles follow the expected behaviour described by the Epstein-Plesset model. .
